# Comment on “Comparison the Diameter of the Urethral Meatus Before and After Circumcision and Evaluation of Urethral Stenosis”

**DOI:** 10.1177/2333794X241277387

**Published:** 2024-09-17

**Authors:** Stephen Moreton, Brian J. Morris

**Affiliations:** 1 CircFacts, Great Sankey, Warrington, Cheshire, UK; 2 University of Sydney, Sydney, NSW, Australia

The relationship, if any, between male circumcision and meatal stenosis (MS) has attracted much attention, with at least 30 studies reporting widely variable results.^1^ A problem is the lack of a standardized, objective diagnostic test, with researchers resorting to very different, sometimes subjective, methods leading to divergent results.^2^

The recent contribution by Hariri et al.^3^ suffers from the same problem. The authors investigated meatus size and risk of MS and found that boys with a smaller meatus were more likely to be diagnosed with MS by their method. This method consisted of measuring what they considered to be the meatus diameter with a ruler. This raises several issues. One is that the meatus is not circular, but elliptical, and is in fact almost a narrow slit, so what they measured (as depicted in their Figure 1) was not a diameter, but the length of the slit, meaning that they only looked at one dimension.

More concerning is their measurement method with a ruler and the result reported to the nearest millimeter. What was the margin of error? For example, was it ± 1 mm? Or was it ± 0.5 mm? This was not stated, which raises concerns about the error margin of their measurements and how this might affect their statistical analysis.

More concerning still is the likelihood of distortion of the meatus during measurement. It is normal for the foreskin of infant boys to be tight and non-retractable, and to have some adhesion to the underlying glans.^4^ Only 7.9% of boys under 3 months of age have a completely visible meatus.^5^ It therefore follows that to perform their measurements on uncircumcised boys, Hariri et al had to retract foreskins that were tight and partially adhering to the glans. Doing so will apply tension to the meatus, stretching it as the frenulum is pulled downwards. Consequently, the measurements will tend to err on the high side.

As the frenular artery was said by Hariri et al to have been ligated during circumcision, it may be assumed that the frenulum was absent when the meatus was measured. This, and the fact that there was no longer a need to retract a tight and adhering foreskin, meant that the meatus could now relax. Any post-circumcision measurement will therefore be a truer representation of the actual dimensions of the meatus. The error introduced need only be slight to have a significant effect. An additional 1 mm turns a 3 mm measurement into a 4 mm one, which is a 33% increase, or a 6 mm meatus measurement into a 7 mm one, a 17% increase. The effect is therefore greater the smaller the meatus, which is what Hariri et al observed.

Hariri offer no biological explanation for why a smaller meatus should be more susceptible to stenosis. Here we have offered a simple explanation for their finding based on measurement bias. Furthermore, the MS rate reported by Hariri et al (10.3%) is very high, casting further doubt on its reliability. A systematic review and meta-analysis found that the great majority of studies reported much lower values: more than half found meatal stenosis prevalence to be <1%, and overall risk of MS in circumcised males was 0.656%.^1^ The review emphasized that diagnosis should be based on a reduction in urine flow because of narrowing of the urethral meatus. Those authors also cited a study of boys circumcised as newborns and diagnosed with MS at age 3 to 8 years using a probe and upward deflection of urinary stream, which found that the appearance of stenosis was an illusion, being instead a “meatal web.” We therefore conclude that the apparent increased risk of MS in boys with a small meatus reported by Hariri et al may be an artefact of the diagnostic method they used.
